# Autophagy: a novel target in order to overcome drug resistance in pancreatic adenocarcinoma

**DOI:** 10.1098/rsob.240412

**Published:** 2025-10-29

**Authors:** Bahareh Shateri Amiri, Mehrasa Naserranjbar, Fatemeh Aliabadi, Alireza Hejrati, Lina Hejrati

**Affiliations:** ^1^ Department of Internal Medicine, Hazrat-e Rasool General Hospital, Iran University of Medical Sciences, Tehran, Iran; ^2^ Department of Medicine, Iran University of Medical Sciences, Tehran, Iran

**Keywords:** pancreatic cancer, pancreatic adenocarcinoma, drug resistance, autophagy, regulating factors

## Abstract

Typically, unchecked pancreatic cell proliferation results in the development of pancreatic cancer, which has the potential to spread to other bodily organs. About 90% of instances of pancreatic cancer are pancreatic adenocarcinomas. About 10–20% of pancreatic carcinomas are resectable and potentially curable, and the 5-year survival rate is only 4%; as a result, the majority of pancreatic cancer treatments are palliative in nature. Surgical resection is the only curative treatment; however, because of late diagnosis, the majority of patients appear at an advanced stage, and only a small percentage (10–20%) of them are candidates for surgery. Due to pancreatic cancer’s strong resistance to practically all chemotherapeutic drugs and conventional radiotherapies, conventional radiation and chemotherapies have little effectiveness in extending patients’ overall life. A lot of scientific studies, however, frequently use the metaphorical term ‘double-edged sword’ to indicate how autophagy plays a different function in cancer. The use of autophagy inhibitors is thought to be advantageous in combining antineoplastic drugs to improve the sensitivity of cancer cells to therapeutic compounds that activate autophagy. In this review, we aim to look into autophagy along with searching for the most effective strategy in order to treat pancreatic adenocarcinoma with the least drug resistance.

**Figure:**
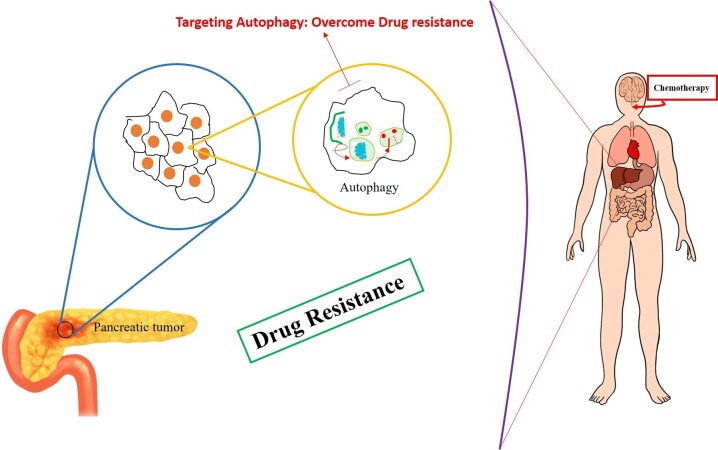

## 1. Introduction

Since autophagic pathways are being targeted by autophagy inhibitors like chloroquine (CQ) and hydroxychloroquine (HCQ), which have previously received clinical approval, it is possible to find and create more advanced and potent cancer therapy strategies. When exposed to medicines or radiation, cancer cells begin to engage in autophagy to protect themselves. Therefore, there is a lot of interest in finding ways to limit treatment-induced autophagy in order to increase the effectiveness of cancer therapies [1]. The use of autophagy inhibitors is thought to be advantageous in combining antineoplastic drugs to improve the sensitivity of cancer cells to therapeutic compounds that activate autophagy [2,3].

A lot of scientific studies, however, frequently use the metaphorical term ‘double-edged sword’ to indicate how autophagy plays different functions in cancer. These investigations mainly rely on genetically modified mouse models (GEMM) and identification of DNA mutations, which permitted the designation of various ATG genes as either ‘tumour suppressors’ or ‘oncogenes’ since they directly affect the biochemical control of autophagy [4,5].

Numerous substances, including mTOR inhibitors, kinase inhibitors, natural products and antiangiogenic medicines, have been discovered to induce cytoprotective autophagy in preclinical and clinical trials as well as numerous therapeutic treatments [6,7]. Phosphoinositide 3-kinase PI3K-Akt-mTOR is one of the primary autophagy regulators, and other signalling pathways and molecules have also been implicated in controlling drug-induced autophagy that affects the effectiveness of anti-cancer therapy. DNA damage brought on by the anti-cancer treatment may be a key factor in the start of autophagy signalling cascades that boost DNA repair levels and support cellular survival [8]. In hepatocellular carcinoma cell lines, research has shown that autophagy is a common cytoprotective response to DNA damage brought on by chemotherapy agents such as cisplatin, BO-1051 and doxorubicin (DOX) [9]. High mobility group box 1 (HMGB1) is linked to cancer’s telltale signs. Numerous cancer cells, including osteosarcoma, lung adenocarcinoma, neuroblastoma and ovarian cancer, have been shown to be protected against multiple chemotherapeutics, such as DOX, cisplatin and etoposide, by autophagy-associated HMGB1 [10]. In human lung adenocarcinoma, HMGB1-mediated autophagy via the mitogen-activated protein kinase (MEK)/extracellular signal-regulated kinase (ERK) signalling pathway enhances docetaxel resistance [11]. In leukaemia, HMGB1 release enhances tumour resistance to chemotherapy and is a critical regulator of autophagy [12].

Additionally, after vincristine, a medication that targets the microtubules, was administered to gastric cancer cells, HMGB1 was released into the extracellular space to prevent cancer cells from dying by inducing the transcription of Mcl-1 [13]. Another signalling route, the VEGF-C/NRP-2 axis, activates autophagy by suppressing mTOR complex 1 activity, which improves cancer cell survival when used as a therapeutic strategy [14]. Some ovarian cancer cells exhibit cisplatin resistance due to protective autophagy that is controlled by PP2Ac and ERK [15,16]. In the therapeutic use of CA-4 for different malignancies, the autophagic response controlled by the JNK-Bcl-2 pathway contributes to limiting the anti-cancer efficacy and toxicity. Therefore, by inhibiting autophagy, a JNK inhibitor or a Bcl-2 inhibitor (ABT-737) might encourage apoptosis induced by CA-4 [17]. In addition to enabling the creation of new cancer treatments, effective targeting of these pathways may be used in therapy to make cancer cells resistant to cellular inhibitors such as cell death, cell proliferation and tumour angiogenesis.

It is crucial to determine if autophagy is necessary for cancerous cells to overcome metabolic and energy stress during carcinogenesis or, on the contrary, whether autophagy (and autophagy-associated cell death) is a crucial step to halt carcinogenesis. The term ‘oncophagy,’ which refers to the intimate relationship between cancer biology and treatment and autophagy, has recently been developed to characterize the function of autophagy in cancer [18].

Typically, unchecked pancreatic cell proliferation results in the development of pancreatic cancer (PC), which has the potential to spread to other bodily organs. About 90% of instances of PC are pancreatic adenocarcinomas, and occasionally ‘pancreatic cancer’ is used exclusively to describe this subtype [19]. Gemcitabine, nab-paclitaxel and FOLFIRINOX are examples of modern chemotherapeutic drugs that have the ability to quickly confer resistance in pancreatic tumour cells. Therefore, in order to treat this dreadful condition, it is essential to develop more effective medicines. Therefore, improving the sensitivity of PC to gemcitabine may improve the prognosis of this fatal disease [20]. In PC, autophagy suppression promotes apoptotic cell death in response to gemcitabine and other stimuli. Remarkably, expanding research has demonstrated that preventing autophagy in cancer treatment can eliminate drug resistance [5]. In this review, we attempt to investigate autophagy in order to overcome the drug resistance in pancreatic adenocarcinoma.

## 2. Pancreatic adenocarcinoma

One of the deadliest types of cancer, pancreatic ductal adenocarcinoma (PDAC) has a 6- to 8-month life expectancy upon diagnosis and a 5-year survival rate of fewer than 5% [21]. Despite the increased survival rates seen in a number of malignancies, such as breast, prostate and colon, PC patients’ overall survival rates have not changed much over the last 30 years [22]. PC has a case-to-fatality ratio of 1, which is greater than that of lung cancer (0.84) and brain tumours (0.68). After diagnosis, the typical survival time is fewer than six months, and the 1 year survival rate is less than 10% [23]. The majority of patients with PC present late and have either locally progressed or metastatic disease, which makes diagnosis difficult [24].

Gene mutations are the fundamental cause of pancreatic carcinogenesis, particularly those involving TP53 (tumour protein p53), SMAD4 (SMAD family member 4), BRCA2 (BRCA2 DNA repair associated) and KRAS (KRAS proto-oncogene, GTPase) [25]. KRAS mutations, which are a major cause of PC, were present in 99% of patients. In mice, pancreatic intraepithelial neoplasia (PanIN) can form and proceed to PDAC with notable stromal responses when KRASG12D mutations are pushed into the pancreas [26].

About 10−20% of pancreatic carcinomas are resectable and potentially curable, and the 5 year survival rate is only 4%. Hence, the majority of PC treatments are palliative in nature [23]. Surgical resection is the only curative treatment; however, because of late diagnosis, the majority of patients appear at an advanced stage, and only a small percentage (10−20%) of them are candidates for surgery. Surgery of PDAC patients needs adjuvant chemotherapy with or without radiation to achieve a 5 year survival rate of 15−25% because of the high recurrence risk [27]. In comparison to observation alone, a recent research found that postoperative gemcitabine medication dramatically reduced the risk of recurrent illness following full resection of PC [28].

However, further research is required to determine which patient population may benefit most from the use of chemo-radiotherapy in the neoadjuvant situation. There have been some encouraging results demonstrating a further increase in survival [24]. For the treatment of pain and obstructive jaundice, strictly palliative approaches include nerve blockades, analgesic medications and bypass surgery [23].

Conventional treatment is ineffective against PCs since they are frequently already invasive and metastatic [29]. Due to the PC’s strong resistance to practically all chemotherapeutic drugs and conventional radiotherapies, conventional radiation and chemotherapies have little effectiveness in extending patients’ overall life [30]. For PC, gemcitabine (2',2'-difluorodeoxycytidine) is the usual first-line treatment [31]. Despite the fact that gemcitabine has considerable positive effects on patients, the response rate, prognosis and progression-free, disease-free and overall survival all show only minor improvements. Therefore, any method that increases PC’s sensitivity to gemcitabine may help the prognosis of this devastating condition [32].

## 3. Drug resistance

Antineoplastic resistance, which is often confused with chemotherapy resistance, is the ability of neoplastic (cancerous) cells to survive and grow in the face of anti-cancer therapies [33]. Cancers can develop resistance to multiple drugs in some cases, which is known as multiple drug resistance.

Gemcitabine and other therapeutic medications are successful in treating patients with advanced and metastatic PC, but the development of gemcitabine chemoresistance significantly restricts the efficacy of this treatment [34]. Gemcitabine has proven to be more effective against PC cells than other chemotherapy agents. The majority of studies on chemoresistance in advanced PC have concentrated on gemcitabine as investigation into the impact of other medications is still in its infancy. Unknown is the underlying mechanism that leads to gemcitabine resistance. The development of chemoresistance to gemcitabine involves a number of transcription factors, including enzymes and signalling pathways involved in nucleoside metabolism [35].

Failure of antineoplastic treatment has two main causes: cancer cells’ resistance is a result of both acquired resistance following medication exposure and inherent genetic traits, which are founded in the idea of cancer cell heterogeneity [36]. Resistant cells have altered membrane transport, improved DNA repair, abnormalities in the apoptotic pathway, changes to target molecules, proteins and route mechanisms, such as enzyme deactivation [36]. Given that cancer is a genetic illness, two genomic events—genome alterations (such as gene amplification and deletion) and epigenetic changes—underlie acquired treatment resistance. To maintain their survival against antineoplastic medications, cancer cells are continually exploiting a number of tools, including genes, proteins and altered pathways [36].

**Figure 1.**
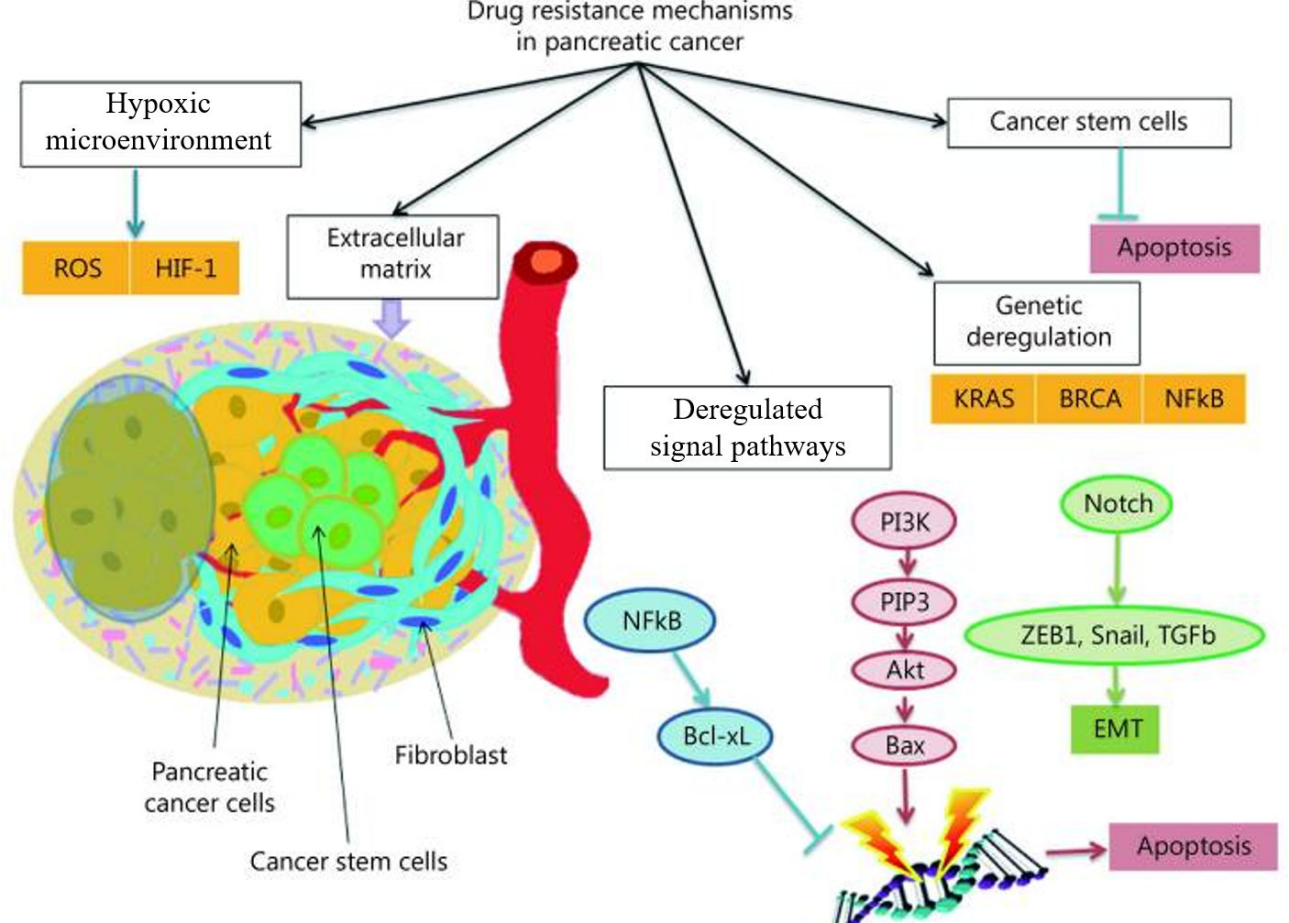
Different drug resistance mechanisms in pancreatic cancer lead to the genetic deregulation of KRAS and BRCA as well as the deregulation of RAS, NFB and PI3K signalling pathways. These mechanisms include the presence of cancer stem cells that are able to undergo apoptosis, tissular hypoxia that raises HIF-1 levels and lowers reactive oxygen species (ROS), and a thick extracellular matrix that prevents the diffusion of chemotherapy [33].

More than 165 genes associated with drug resistance were identified by gene expression microarray analysis carried out in PC cell lines. These genes were implicated in a wide range of cellular processes, such as signal transduction, cell cycle control, antioxidant activity and apoptosis, among others [37] (figure 1). Gemcitabine activates the AMPK/mTOR pathway, raising the expression of AMPK and reducing that of mTOR, leading to cell autophagy, which causes tumour cells to stop growing and undergo apoptosis [38]. Through this pathway, PC cells undergo the drug-related epithelial-mesenchymal transition (EMT), which is induced by ARK5, an AMPK-related kinase. Recently, it was discovered that gemcitabine may sensitize pancreatic cells when ARK5 was inhibited by altering the oxygen levels (normoxia/hypoxia) [39].

### 3.1. Autophagy, pancreatic adenocarcinoma and drug resistance

Autophagy is an autolytic mechanism involved in the lysosomal disposal of damaged organelles, malformed proteins during biosynthesis and non-functional long-lived proteins. Only a fine number of autophagy in cells is involved in keeping up homeostasis under physiological conditions. To date, three main types of autophagy have been described including macroautophagy, microautophagy and chaperone-mediated autophagy. Macroautophagy affects the formation of autophagosomes, double-membrane vesicles. Autophagosomes capture cytoplasmic contents and fuse with lysosomes to create autophagolysosomes. Autophagolysosomes are structures in which cargo substrates are degraded by lysosomal enzymes [40–42]. In microautophagy, cytoplasmic components are directly taken up by lysosomes and degraded [43–45]. Therefore, three types of autophagy count on active lysosomes to digest intracellular cargo.

Induction of autophagy is triggered by a variety of intracellular and extracellular stimuli like total amino acid and serum depletion, which potently induce high levels of autophagy and oxidative stress that induce autophagy to recycle damaged organelles (such as mitochondria) and clear protein aggregates, and nutritional deficiencies, such as rapamycin and CCI-779, as mTOR inhibitors [46–48]. When cells are induced by these intracellular and extracellular stimuli, ATG13 anchors ULK1 to preautophagosomal structures (PAS), on which almost all autophagy-associated (Atg) proteins are hierarchically assembled. It has been reported to be a key cytoplasmic site for vacuolar targeting and autophagosome formation [49–51]. As a reservoir structure for ATG protein recruitment, PAS plays an important role in the induction of autophagy [51,52]. Under autophagy-inducing conditions, the ULK1/Atg1 functional unit (including ULK1, ATG13, FIP200 and ATG101) functions as an autophagy induction complex, and ATG13 is critical for PAS localization of ULK1 (Atg1 in yeast). When ATG13 and ULK1 target PAS, all these multiple ATG proteins primarily bind and localize to PAS to initiate autophagy [49–51]. Afterwards, other active units, containing the ULK1 complex, the PI3K complex, the ATG9A system, the ATG12 binding system and the LC3 binding system, hierarchically align to the PAS and participate in autophagosome assembly and formation [53].

The attachment and integration of autophagosomes with lysosomal membranes require the transport of mature autophagosomes to the perinuclear area for autophagosome–lysosome fusion [54]. Autophagosomes are formed arbitrarily across the cytoplasm, considering lysosomes are found mainly in the perinuclear area. Consequently, when mature autophagosomes are produced, they must be delivered to the perinuclear area [55]. By the time autophagosomes reach the perinuclear area, they immediately fuse with lysosomes to form autophagolysosomes. When an autophagosome fuses with a lysosome to form an autophagolysosome, many enzymes within the lysosome, such as lysosomal hydrolases, the inner membrane of the autophagosome and macromolecules are derived from the cytoplasm. Proteins and organelles within autophagosomes are converted to amino acids or peptides for reuse by the cell [55].

In this regard, it is confirmed that many of the proven risk factors of PDAC can upregulate autophagy. For example, in a chemical carcinogenic scenario where azamatrine was given to Wistar rats, the autophagy capacity of pancreatic premalignant atypical acinar nodule cells is similarly elevated [56]. In order to shield pancreatic ductal cells from damaged organelles or gene changes during tumorigenesis, it was later demonstrated that brief exposure to cigarette smoke extract or smoking compounds, such as 4-(methylnitrosamino)-1-(3-pyridyl)-1-butanone/NNK, would trigger autophagy by activating AMPK [57]. Long-term exposure to smoking chemicals, however, inhibits autophagy and apoptosis, which encourages the development of tumours in mice [57]. Similarly, autophagy is suppressed by MAGEA6 (MAGE family member A6; a bioenergy sensor), which in turn speeds up the onset of PDAC in mice [58]. In a chemical carcinogenic scenario, where azamatrine was given to Wistar rats, the autophagy capacity of pancreatic premalignant atypical acinar nodule cells is similarly elevated [56]. In order to shield pancreatic ductal cells from damaged organelles or gene changes during tumorigenesis, it was later demonstrated that brief exposure to cigarette smoke extract or smoking compounds, such as 4-(methylnitrosamino)-1-(3-pyridyl)-1-butanone/NNK, would trigger autophagy by activating AMPK [57]. Long-term exposure to smoking chemicals, however, inhibits autophagy and apoptosis, which encourages the development of tumours in mice [57]. Similarly, autophagy is suppressed by MAGEA6 (MAGE family member A6; a bioenergy sensor), which in turn speeds up the onset of PDAC in mice [58].

It is interesting to note that one of the most recent developments in this area is the comprehension of the connection between autophagy and apoptosis in human tumours. It is widely known that autophagy and apoptosis interact in human malignancies; for instance, autophagy is upregulated when the circular RNA circ-TICRR is silenced, which causes cancer cells to undergo apoptosis [59]. It appears that cytoprotective autophagy can also occur in tumour cells [60], and reduction of supporting autophagy promotes mitochondrial-mediated apoptosis in cancer [61], despite the fact that autophagy has been demonstrated to accelerate apoptosis. More significantly, autophagy influences the EMT and other metastasis-related processes [62]. Targeting autophagy can have conflicting effects on cancer because of its perhaps opposing roles in either accelerating [63] or inhibiting [64] metastasis.

Autophagy is a two-edged sword that, depending on the situation, can either encourage or prevent the survival and spread of cancer cells. Thus, it is difficult to address autophagy therapeutically in human malignancies. Autophagy’s impact on other cell death pathways is occasionally connected to its role in cancer cell survival. The activation of AMPK to trigger autophagy is mediated by SIRT3 expression, and this autophagic stimulation can increase ferroptotic cell death [65]. In accordance with these findings, ferroptosis can be induced when autophagy has a pro-death role [66]. Ferritin degradation that is dependent on autophagy can also promote ferroptosis sensitivity in cancer cells [67]. On the other hand, autophagy occasionally helps cancer cells survive. Melanoma growth is hampered by the reduction in autophagy caused by MAPK/p38 [67,68], and listeria monocytogenes’ anti-cancer actions are explained by the interruption of autophagy [69]. Anti-tumour agents can occasionally encourage tumour regression by suppressing pro-survival autophagy [70]. However, pro-death effects are not necessarily mediated via autophagy control and activation by anti-cancer medicines.

Autophagy has a complicated function in controlling inflammation at the beginning of PC. The precursor of PanIN is a pathological alteration known as acinar-ductal metaplasia (ADM), which occurs when oncogenic signalling is activated (e.g. gene mutation and inflammation). ADM is formed by endosplasmic reticulum stress, mitochondrial failure and defective protein synthesis when pancreatic epithelial cells (Pdx1-Cre;atg7fl/fl) have conditional deletion of Atg7, which causes severe acinar cell degeneration [71]. By controlling cell death signals, Atg7 deficiency in the pancreas (Pdx1-Cre;atg7fl/fl) also causes inflammation and fibrosis in mice [72], suggesting a possible role for ATG7-mediated inflammatory regulation in the development of PC. Because autophagy increases TBK1 (TANK binding kinase 1)-mediated dysplasia, it contributes to carcinogenesis in KRAS-driven PC [73,74]. However, because enhanced autophagy decreases TBK1-mediated inflammation in mice, pancreatitis and dysplasia are made worse by autophagy inhibitors or Atg5 deletion (Pdx1-Cre;atg5fl/fl [74]). Consequently, autophagy has two roles in the process of inflammation-induced pancreatic carcinogenesis, as will be discussed later. By regulating proliferation, invasion and metastasis, metabolism, cell death or immunity, autophagy also plays two roles in the development of PDAC. In the basal state, PC cell lines and patient tumour tissues exhibit a high amount of autophagy in established PC [75]. Early research indicates that increased autophagy contributes to the progression of PDAC because genetic (e.g. atg7-/- or hmgb1-/-) and pharmacological (e.g. using CQ) inhibition of autophagy results in the production of reactive oxygen species (ROS), increased DNA damage and metabolic dysfunction, which ultimately prevents the development of PC *in vitro* and *in vivo* [75,76]. According to recent research, increasing autophagy may also slow the progression of PDAC.

Numerous studies show that in a variety of tumour types, increased autophagy not only increases cell survival but also increases tumour treatment resistance. Important hints are starting to surface, despite the fact that the exact processes by which autophagy encourages tumour treatment resistance remain unclear. In response to capsaicin, ataxia telangiectasia mutated (ATM)-mediated activation of DNA-dependent protein kinase catalytic subunit (DNA-PKcs) and poly(ADP-ribose) polymerase-1 has been demonstrated to directly induce an autophagy-regulated DNA damage response [77]. Autophagy also triggers an increased DNA damage response through the homologous recombination repair pathway, a key mechanism for mending double-strand breaks [78]. Furthermore, epirubicin-induced stimulated autophagy can lead to EPI resistance by increasing drug efflux through P-glycoprotein, encoding MDR genes that lower intracellular drug concentrations, and downregulating the NF-κB signalling pathway, which lowers the rate of apoptosis [79]. In order to increase intracellular drug efflux, endosplasmic reticulum stress-triggered autophagy also upregulates multidrug resistance-associated protein 1 (MRP1), another important protein encoded by MDR genes [80]. During temozolomide therapy, autophagy also regulates aldehyde dehydrogenase 1A3, a detoxifying enzyme that promotes acquired drug resistance [81]. Induced autophagy releases HMGB1, a prototypical damage-associated molecular pattern protein that enhances treatment resistance in lung cancer [82], colorectal cancer [83] and ovarian cancer [84]. Therefore, autophagy-mediated drug resistance is a complex mechanism that includes gene repair, cytoplasmic material renewal, changes in drug metabolism and concentration and modifications in the expression or activity of important proteins.

## 4. Autophagy: a novel target in pancreatic cancer

### 4.1. Regulators of autophagy in pancreatic adenocarcinoma

Various factors are known to regulate autophagy in cancer, including BECN1, MAPK, (PI3K)-AKT, AMP-activated protein kinase (AMPK) and ATG proteins. Significantly, non-coding RNAs modulate the chemoresistance pathways and are the main regulators of autophagy. Given the dual oncogenic and oncogene-suppressive properties of autophagy, aberrant autophagy status can accelerate or inhibit drug resistance in cancer, and therefore, the only way to overcome chemoresistance is rational targeting of autophagy. Autophagic regulation is a complex process; however, strong evidence suggests that LKB1-AMPK-mTOR, PI3K-Akt-mTOR and p53 are the major upstream regulators of the autophagic pathway. Here, we will specially investigate autophagy regulation in PDAC in detail.

#### 4.1.1. Positive regulators

##### 4.1.1.1. KRAS

An increase in autophagic flux and a reduction in glycolytic and mitochondrial activity were caused by KRAS suppression and ERK inhibition. This suggests that ERK inhibition increases the dependency of PDAC on autophagy, and that successful PDAC therapy requires pharmacologic inhibitors that operate against both ERK MAPK and autophagy [8,5]. Interestingly, a novel approach to treating tumours in autophagy-mediated drug resistance may involve either a decrease in autophagy flux (limiting autophagy for survival) or an increase in autophagy flux exceeding the threshold (autophagic cell death). The increase of VMP1, a protein required for the production of autophagosomes, may be one way whereby oncogenic KRAS positively controls autophagy in PDAC [86]. The Hedgehog pathway regulates GLI3, a transcription factor that also stimulates the transcription of VMP1, and RNAi studies have demonstrated that VMP1 is required for KRAS to trigger and sustain autophagy [86].

##### 4.1.1.2. SNHG14

RNAs longer than 200 nucleotides, known as long non-coding RNAs, may control autophagy in cancer cells [87]. Particularly, the small nucleolar RNA host gene 14 (SNHG14) is expressed more strongly in PDAC compared with normal tissue and plays a role in the advancement of numerous cancer types [88]. It has been demonstrated that the negative autophagy regulator microRNA miR-101 interacts with SNHG-14. This interaction may lower the levels of miR-101, which would induce autophagy and make PDAC cells more resistant to gemcitabine [89].

### 4.1.2. Negative regulators

#### 4.1.2.1. UBL4A

The two main elements of the lysosomal membrane are lysosome-associated membrane protein-1, LAMP1 and LAMP2, which regulate the development of autophagosomes [90]. According to additional data, PDAC metabolic alterations are what activate the transcription of the lysosome biogenesis gene [91]. UBL4A, a tumour suppressor that mediates cell death in response to DNA damage and a protein folding chaperone, has been discovered to directly interact with LAMP1 in a recent research [89]. Because of the autolysosome accumulation and lysosomal dysfunction seen in cells with greater UBL4A levels, it is assumed that the interaction between UBL4A and LAMP1 disrupts lysosome function and impedes autophagic breakdown. Additionally, the investigation of UBL4A expression in 69 patients with PDAC showed that individuals with greater levels of UBL4A mRNA in PDAC tissue have superior survival rates [92], possibly because they also have lower levels of autophagy [89].

#### 4.1.2.2. Apoptosis

Type I programmed cell death, also known as apoptosis induction, can occur internally in response to cellular stress or extrinsically as a result of the ligation of a death receptor (such as Fas/CD95) at the plasma membrane. These internal stresses, which include DNA damage, lead to the overexpression of proapoptotic proteins, which permeabilize the mitochondrial membrane, deplete the mitochondrial membrane potential and release apoptogenic factors. Once released from the mitochondria, these substances can either directly induce apoptosis or do so indirectly by activating the family of cysteine proteases known as caspases. Bax and other members of the Bcl-2 family of proteins, as well as other regulators like p53, regulate the intrinsic mitochondrial pathway. A caspase protein called caspase 3 is activated by caspases 8 and 9 and then facilitates substrate cleavage and cell apoptosis [93,94].

### 4.2. Current therapeutic candidates targeting autophagy in pancreatic adenocarcinoma

According to recent research, either autophagy stimulation or inhibition might assist patients therapeutically depending on the situation, and the design and synthesis of autophagy modulators may offer unique therapeutic tools that could ultimately result in new cancer treatment approaches. When injected into mice [2], primary PC cell lines with Atg5 knockdown showed an enhanced propensity for invasiveness and metastasis. Lower levels of Atg5 were linked to tumour spreading and a shorter survival time in human PDAC samples that were also under observation [95]. There is a rise in resistance to chemotherapy, radiation, certain anti-cancer drugs and targeted treatments when there are defects in apoptosis. Therefore, when treating tumours that are resistant to apoptosis by anti-cancer medicines, inducing autophagic cell death may be the best course of action (e.g. chemotherapy, radiation) [2]. A hallmark of metastasis and a poor patient prognosis in PC, autophagy, is inhibited by protein kinase C-delta (PKC_) via activation of transglutaminase 2 (TG2). This was identified by Ozpolat *et al.* [96]. The PKC /TG2 axis was shown to be a possible novel therapeutic target by inducing autophagy [96]. Rottlerin, a PKC delta inhibitor, also caused PC to undergo intrinsic and extrinsic apoptosis. However, rather than PKC, eukaryotic elongation factor-2 kinase (eEF2K) constituted the basis for this action. However, Rottlerin enhanced eEF2ubiquitin-mediated K’s proteasomal degradation and decreased its expression [97]. The following sections describe the most famous and effective treatments of PDAC, and we will investigate the best way of targeting autophagy in each scenario separately to find out whether it is better to inhibit or enhance this pathway, and how to do so in each condition (table 1).

**Table 1.** Chemotherapeutic agents that reduce drug resistance by modifying autophagy.

name of the autophagy modifying agent	combined with…	pathway of action	effectivity	model of study	clinical trials	results	reference
canabinnoids	gemcitabine	stimulating the mRNAs of both CB1 and CB2 via an NF-κB-mediated mechanism	causes autophagy in the cancer cells	morthotropic model	SR1 as an anti-cancer agent	the combination of gemcitabine and cannabinoid receptor agonists in the treatment of pancreatic cancer enhanced intracellular production of ROS, resulting in antiproliferative effects	[98]
hydroxychloroquine	trametinib	the RAF-MEK-ERK pathway with a MEK inhibitor along with the concurrent use of an autophagy inhibitor such as hydroxychloroquine	— inhibition of autophagy — combining HCQ with gemcitabine and nab-paclitaxel (GA) in mature PC patients enhances their efficacy	molecular profiling studies (cell model)	FDA approved	the real-life data regarding KRAS-mutated PDAC patients who received treatment with the MEK inhibitor trametinib combined with hydroxychloroquine after experiencing disease progression are consistent with the preclinical data, pointing to the clinical benefits of this regimen	[99]
genistein	5-FU	Bcl2 and beclin-1	— enhance the anti-cancer effects of chemotherapy drugs (such as gemcitabine, cisplatin and oxaliplatin) — enhance 5-FU-induced autophagy promotes autophagy	— cell lines and reagents using the MIA PaCa−2 human pancreatic cancer cell line — animal experiment using MIA PaCa-2 human pancreatic cancer cells in a subcutaneous xenograft model	Not yet	— genistein enhances the anti-tumour effect of 5-FU *in vitro*	[100]
	cisplatin	inactivation of the NF-κB signalling pathway by genistein and Akt activation	enhance the anti-tumour effect of cisplatin	orthotopic tumour model	not yet	primary pancreatic cancer with de novo and acquired resistance to chemotherapeutic drugs such as cisplatin could be reversed by genistein pretreatment. The marked ability of genistein and cisplatin within therapeutic range to induce apoptosis *in vitro* and greater anti-tumour activity *in vivo* suggests that this is an active and attractive regimen for patients diagnosed with pancreatic adenocarcinoma, similar to genistein and gemcitabine	[101]
	gemcitabine	inactivation of NF-κB signalling	enhance the anti-tumour effect of cisplatin in orthotopic tumour model	orthotopic tumour model	not yet	dietary genistein may block multiple intracellular signalling pathways that are known to confer a high degree of chemoresistance by pancreatic cancer cells, thereby abrogating either de novo or acquired chemoresistance	[102]
suberoylanilide hydroxamic acid (SAHA) (histone deacetylase (HDAC) blockers)	gemcitabine	akt, mTOR and ribosomal protein S6 kinase B1 (RPS6KB1, also known as p70S6K) axis and promotion of the ERK1/2 pathways — SAHA induced caspase-3 activation and apoptosis through the mitochondria_cytochrome *c*-mediated apoptotic pathway	— stimulate autophagy in cancer — enhance the sensitivity of PC cells to gemcitabine	cell lines	not yet	HDAC inhibitors induced caspase activation and apoptosis caspase-independent cell death induced by HDAC inhibitors	[97,103]
GGPTase-I inhibitors (GGTI) and farnesyltransferase inhibitors (FTIs))	radiotherapy	— block GGT-positive tumours from accessing the cysteine in extracellular glutathione — alternatively prenylation of KRAS and NRAS proteins	sensitize tumours to chemotherapy	a phase i study	L-778,123 as an anti-cancer agent	inhibition of GGT is a new approach to overcoming drug resistance in tumours. Inhibition of GGT several hours prior to the administration of chemotherapy would not only block the access of GGT-positive tumours to cysteine from extracellular GSH but would also inhibit GGT in the kidney, resulting in glutathionuria and a rapid reduction in the levels of cysteine in the blood. As a result, GSH concentrations in both GGT-positive and GGT-negative tumours would decrease and the tumours would be sensitized to the therapy	[104,105]
JC-TH-acetate-4 (JCTH-4)(C-1 analogue of 7-deoxypancratistatin)	used alone in chemoresistant pancreatic cancer	induce apoptosis selectively in BxPC-3 and PANC-1 cells by targeting the mitochondria; it dissipated mitochondrial membrane potential, caused release of apoptogenic factors and in isolated mitochondria, increased the generation of reactive oxygen species	selectively induced autophagy in pancreatic cancer cells while normal human fetal fibroblasts were markedly less sensitive to JCTH-4	cell culture (pancreatic cancer cells (BxPC-3, PANC-1))	not yet	— JCTH-4 selectively induces cytotoxicity in pancreatic carcinoma cells — JCTH-4 selectively induces apoptosis in pancreatic carcinoma cells — JCTH-4 selectively induces apoptosis by targeting mitochondria in pancreatic carcinoma cells	[93]
ascorbate	both alone and in combination with FOLFIRINOX	induce oxidative stress	autophagy can be induced by ROS, such as H_2_O_2_, and may aid in cell death	— cell culture and viability assay — phase I/IIa clinical trial	phase I/IIa clinical trial	a phase I trial by Hoffer *et al.* [106] treating 24 terminal cancer patients found that IVC dosed at 1.5 g kg^−1^ 3× weekly was free of significant toxicity, and unexpectedly, two patients had stable disease	[106–108]
overexpression of miR-29a	gemcitabine	— miR-29a downregulates critical autophagy proteins TFEB and ATG9A and knockdown of TFEB and ATG9A results in decreased autophagosomal–lysosomal fusion — TGF-β1 downregulates miR-29 expression in other cell types through SMAD2/3/4 complex	— inhibits autophagy flux in pancreatic cancer cells — miR-29a inhibits autophagosome–lysosome fusion — inhibit anchorage independent growth, invasion and migration of cancer cells — overexpression of miR-29a makes chemotherapeutic-resistant pancreatic cancer cells susceptible to gemcitabine	cell culture RNA purification	not yet	miR-29a functions as a potent autophagy inhibitor, sensitizes cancer cells to gemcitabine and decreases their invasive potential	[109]

#### 4.2.1. Gemcitabine

One of the traditional chemotherapeutic medications used to treat PDAC is gemcitabine, as was previously noted; however, patients frequently only respond partially because of resistance. Inhibiting autophagy can make PDAC cells more sensitive to gemcitabine [89]. When used as the first-line treatment for individuals with advanced PC, gemcitabine causes autophagy in the cancer cells. In PC, autophagy suppression promotes apoptotic cell death in response to gemcitabine and other stimuli [94]. Apoptotic cell death is allegedly caused by gemcitabine-induced VMP1-mediated autophagy, according to other research [110]. Nevertheless, experimental pancreatitis activates zymophagy, a new selective autophagy mechanism, which is regulated by the VMP1-USP9x-p62 pathway and stops pancreatic cell death. Gemcitabine also has a stronger cytotoxic impact when coupled with cannabis, which causes autophagic cell death in pancreatic tumour cells via ROS. In fact, by activating the endoplasmic reticulum stress pathway in PC cells, cannabinoids also promote apoptosis [94].

#### 4.2.2. Hydroxychloroquine

Most PDACs have higher basal levels of autophagy. Human PC development is inhibited by pharmacological inhibition of autophagy using CQ or genetic inhibition of critical autophagy genes with RNAi. This suggests potential therapy options for PDAC patients employing the CQ-derivative HCQ [111]. It is important to note that the US Food and Drug Administration has previously authorized HCQ, a possible autophagy inhibitor, and that it may work well to modulate autophagy in people with PC [94]. CQ therapy may raise the chance of producing resistant cancer cell clones with increased aggressiveness, which has substantial therapeutic implications. Monoallelic deletion of Atg5 resulted in resistance to the autophagy inhibitor CQ and a greater metastatic spread [89].

#### 4.2.3. Genistein

When examined utilizing *in vitro* and *in vivo* models, the isoflavone genistein, which is produced from soy, has a variety of biological actions against different forms of cancer. Genistein can enhance the anti-cancer effects of chemotherapy drugs (such as gemcitabine, cisplatin and oxaliplatin) via modifying the apoptotic pathway, according to many studies. Furthermore, current research shows that genistein promotes autophagy [100]. By drastically changing the expression of two crucial molecules, Bcl2 and beclin-1, which control autophagy, Suzuki *et al.* [100] showed that the combination of 5-fluorouracil (5-FU) and genistein caused autophagic cell death. An anti-apoptotic protein called Bcl2 is overexpressed in many cancer types, and by preventing chemotherapy-induced apoptosis, it aids in the development of treatment resistance [100]. By attaching to the BH3 domain of beclin-1 and inhibiting the autophagy-promoting beclin-1 vacuolar protein sorting 34 complex, Bcl2 is also implicated in inhibiting autophagy. Mutant beclin-1 proteins that do not bind to Bcl2 have been shown to cause excessive cellular autophagy and death in the absence of autophagic cues [112]. Therefore, a plausible molecular explanation for the observed induction of autophagic cell death with the combination of 5-FU and genistein is the downregulation of Bcl2 and upregulation of beclin-1 revealed here [100].

#### 4.2.4. Histone deacetylase blockers

Aside from these epigenetic and genomic alterations, PC also exhibits DNA methylation and histone modifications. Notably, histone deacetylase (HDAC) inhibitors have an impact on autophagic and epigenetic processes. Suberoylanilide hydroxamic acid (SAHA), a potential treatment and HDAC inhibitor, induced autophagy in cancer cells while blocking the Akt/mTOR pathway and inducing the endosplasmic reticulum stress response. Additionally, SAHA made PC cells more sensitive to gemcitabine [97].

#### 4.2.5. Inhibitors of -glutamyl transpeptidase

It has been demonstrated that new, non-toxic inhibitors of -glutamyl transpeptidase (GGT) cause cell death in leukaemia and liver cancer cells that overexpress GGT [113]. As glutathione metabolism and autophagy are activities that PDAC cells depend on for life, this has a bearing on PDAC therapy [89].

#### 4.2.6. C-1 analogue of 7-deoxypancratistatin

Numerous cancer cell types have been demonstrated to be selectively induced cytotoxic by the natural substance pancratistatin (PST). However, its natural source only contains trace amounts of it, and its chemical production has been hampered by several difficulties. By creating JC-TH-acetate-4 (JCTH-4), a C-1 acetoxymethyl analogue of 7-deoxypancratistatin, Dennis Ma *et al.* were able to circumvent these barriers and produce a substance with similar selective anti-cancer action as PST [93]. JCTH-4 (BxPC-3, PANC-1) is a powerful chemotherapeutic against PC cells. By specifically targeting the mitochondria, it was able to induce apoptosis in BxPC-3 and PANC-1 cells. This was accomplished by causing the release of apoptogenic factors, dissipation of mitochondrial membrane potential and an increase in the production of ROS in isolated mitochondria. Furthermore, PC cells were specifically stimulated to undergo autophagy by JCTH-4 [93].

#### 4.2.7. miR-29a

The innovative medicinal drug miR-29a targets PDAC. Small non-coding RNAs called miRNAs are conserved and control post-transcriptional gene expression [32,33]. These tiny molecules are widely expressed in healthy tissue and frequently misregulated in pathological conditions. It has been proposed that restoring the expression of downregulated miRNAs is advantageous when trying to treat cancer [109]. In PC cells, miR-29 is downregulated, and overexpression of miR-29a makes chemotherapeutic-resistant PC cells susceptible to gemcitabine, while also decreasing cancer cell viability and increasing cytotoxicity [109]. The buildup of autophagosomes and autophagy markers, LC3B and p62, as well as a decline in autophagosome–lysosome fusion, are further indications that miR-29a hindered autophagy flow. The autophagy proteins TFEB and ATG9A, which are essential for lysosomal function and autophagosome trafficking, respectively, were also expressed at lower levels as a result of miR-29a. Similar to miR-29a overexpression, autophagy was suppressed by TFEB or ATG9A knockdown. Finally, miR-29a inhibited anchorage-independent growth, invasion and migration of cancer cells [109].

#### 4.2.8. Ascorbate

PC is one of the cancer cell types that are preferentially killed by pharmacological ascorbate (ascorbic acid, vitamin C). H_2_O_2_ production is necessary for cell death. Ascorbate concentration and incubation period affect H_2_O_2_ production. Pharmacological ascorbate concentrations result in extracellular H_2_O_2_ that diffuses into the cells and causes oxidative stress, ultimately leading to cell death [107]. Uncertainty exists on how autophagy affects cellular reactions to oxidative stress. Autophagy can be induced by ROS, such as H_2_O_2_, and may aid in cell death. In contrast, ROS-mediated necrosis may be protected by autophagy. Through a special caspase-independent autophagy route, ascorbate may cause death. Ascorbate-treated PC cells show an increase in LC3-II immunoreactive protein. Pretreating the cells with catalase prevents this rise in LC3-II, indicating that H2O2 is a mediator of the ascorbate-induced activation of autophagy [107].

#### 4.2.9. Treatments targeting TGFB1

Depending on the specific genetic environment, TGFB1 stimulated autophagic flow via SMAD4-dependent or SMAD4-independent mechanisms. By reducing the nuclear translocation of SMAD4, TGFB1-induced autophagy increased proliferation and reduced migration in SMAD4-positive PDAC cells. On the other hand, TGFB1-induced autophagy increased migration and suppressed proliferation in SMAD4-negative cells through controlling MAPK/ERK activation [114].

#### 4.2.10. Targeting ROS

Apoptosis and autophagy are two intracellular processes in which ROS function as signalling molecules [115]. One of the main sources of ROS is mitochondria. ROS toxicity is used by radiation treatment and various types of chemotherapy to kill tumour cells. ROS are produced more when autophagy or mitophagy are inhibited, which causes apoptotic cell death. The initial evidence for autophagic cell death came from the detection of elevated autophagic markers in dying cells. A growing body of research suggests that autophagic cell death occurs when a cell dies with autophagy rather than when an autophagic process kills it [94].

#### 4.2.11. Targeting high mobility group box 1-RAGE

**Figure 2.**
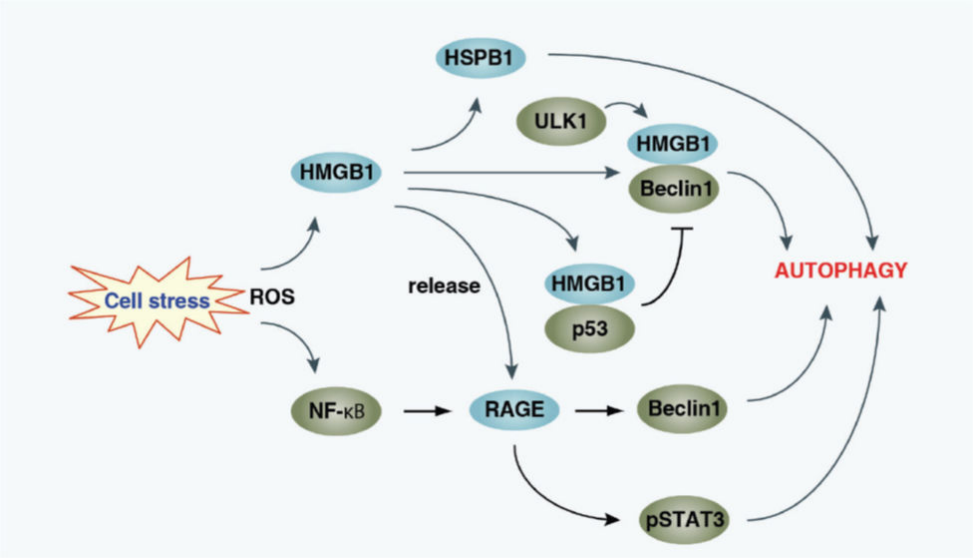
An autophagy sensor under oxidative stress is HMGB1. In the control of autophagy, HMGB1 performs crucial intranuclear, cytosolic and extracellular functions. The expression of HSPB1, which is necessary for the dynamic of mitophagy to govern mitochondrial quality, is regulated by nuclear HMGB1. A protein that binds to Beclin1 is cytosolic HMGB1 [94]. A downstream signal from the ULK1 complex is the HMGB1 Beclin1 complex. The amount of the HMGB1-Beclin1 complex is controlled by the interaction between HMGB1 and p53. Loss of p53 increases the HMGB1-Beclin1 complex, cytosolic translocation of HMGB1 and autophagy. Loss of HMGB1, on the other hand, encourages p53’s cytosolic translocation and prevents autophagy. RAGE is bound to active autophagy by exogenous HMGB1 [94]. ROS, like H_2_O_2_, cause NF-κB to become more active, which then promotes the overexpression of RAGE. By enhancing Beclin1-dependent autophagy and reducing apoptosis, this RAGE overexpression enhances drug resistance and protects pancreatic tumour cells from oxidative stress. Additionally, RAGE is necessary for the phosporylation of STAT3 (pSTAT3) brought on by IL-6 and the subsequent activation of autophagy [94].

Inhibition of HMGB1-RAGE causes an increase in apoptosis and a decrease in autophagy in PC cells. The HMGB1-RAGE pathway performs crucial functions in the activation of autophagy (figure 2) [94].

## 5. Conclusion and outlook

One of the types of cancer with the greatest fatality rates is PDAC. Due to its delayed diagnosis, curative resection is not an option, hence the majority of current treatments for PDAC are focused on chemotherapy and radiation therapy. Unfortunately, these methods are insufficient to change the grim outlook. The fast emergence of medication resistance prevents many individuals with PDAC from benefiting from the improvements in chemotherapy (such as nab-paclitaxel and gemcitabine). Gemcitabine formulations encapsulated in albumin nanoparticles have been tested *in vitro* and *in vivo* to improve therapeutic effectiveness. However, 5-FU, a drug that is frequently used to treat colon cancer because it may enter the DNA and stop cell division, is ineffective in PC, where no appreciable improvement in symptoms or survival time was shown [116]. In clinical investigations, the addition of 5-FU to gemcitabine did not result in any therapeutic advantages over gemcitabine alone, although there was a modest increase in adverse effects (neutropenia, diarrhoea and anaemia) [117]. However, FOLFIRINOX, a combination of various medications (5-FU included), prolonged life for individuals with advanced PC by two months. In several clinical studies, the Topoisomerase I inhibitor irinotecan, which is contained in the drug FOLFIRINOX, was successful in treating this particular kind of cancer [118]. Irinotecan’s liposomal encapsulation would make the therapy of refractory PC more effective. Additionally, utilized in PC9 is paclitaxel combined with human albumin (nab-paclitaxel). In a recent clinical research, it was shown that the combination of nab-paclitaxel and gemcitabine increased patients’ survival times for advanced PC by two months without appreciably raising medication toxicity [119]. Currently, nab-paclitaxel, gemcitabine and FOLFIRINOX are the three clinically most successful treatments. Due to the increased toxicity and significant risk for individuals with advanced illness, this regimen is not appropriate for all patients. It is interesting to note that, depending on the genetic background, tumour stage, etc., autophagy can either be ‘protective’ in certain situations, reducing the effects of various cellular stresses and promoting the unintended survival of malignant cells, or it can potentiate various forms of cell death. In response to gemcitabine and other stimuli, autophagy inhibition in PC enhances apoptotic cell death. Surprisingly, growing research has shown that inhibiting autophagy during cancer therapy helps get rid of drug resistance. However, sometimes it is better to induce this pathway regarding the patient’s condition. Therefore, a deep understanding of autophagy and its relative pathways, as well as its regulators, is necessary in order to achieve the best treatment, especially for PC.

## Data Availability

This article has no additional data.
